# Protective Effect of Recombinant Proteins of *Cronobacter Sakazakii* During Pregnancy on the Offspring

**DOI:** 10.3389/fcimb.2020.00015

**Published:** 2020-01-31

**Authors:** Jia-rong Song, Yan-wen Fu, Ping Li, Ting Du, Xin-jun Du, Shuo Wang

**Affiliations:** ^1^State Key Laboratory of Food Nutrition and Safety, College of Food Science and Engineering, Tianjin University of Science and Technology, Tianjin, China; ^2^Tianjin Key Laboratory of Food Science and Health, School of Medicine, Nankai University, Tianjin, China

**Keywords:** *Cronobacter sakazakii*, maternal immunization, offspring, GroEL, OmpX

## Abstract

*Cronobacter sakazakii* is a food-borne pathogen carried in milk powder that can cause severe bacteremia, enterocolitis, and meningitis in newborns, which can lead to death of newborns. Preventing infection by this pathogen is significant to the health of newborns. Since infants and young children are the main target group of *C. sakazakii*, it is considered that maternal immunity can enhance the protection of newborns. Previous studies showed that two proteins of *C. sakazakii* (GroEL and OmpX) exhibited high expression levels and elicited strong immune reactions, suggesting their potential as vaccine candidates. In this study, GroEL and OmpX were recombinantly expressed in *Escherichia coli* and purified as immunogens to immunize pregnant rats. Three days after birth, the progeny were challenged with *C. sakazakii* to determine the protective effect of maternal immunity on the offspring. The results showed that immunization during pregnancy decreased bacterial load in the brain and blood, reduced brain and intestine damage, and significantly increased specific antibody titers in the offspring. Immunization with the recombinant proteins significantly increased cytokine levels in the serum of the progeny. The group whose mothers were immunized with OmpX produced more IL-4, while the group whose mothers were immunized with GroEL produced more IFN-γ, indicating that the immunogens enhanced the Th2 and Th1 responses, respectively. However, although the immune response was induced by both proteins, only the offspring of the pregnant rats immunized with OmpX or OmpX/GroEL mixture showed delayed death, possibly because immunization with OmpX led to a stronger humoral immune response in the offspring, suggesting that OmpX was a better vaccine candidate than GroEL. This study first reported that exposure to *C. sakazakii* proteins during pregnancy could improve the offspring's ability to resist infection caused by this pathogen.

## Highlights

- OmpX and GroEL are effective vaccine candidates for maternal immunization.
- Immunization during pregnancy decreased the bacterial load in infected offspring.
- Immunization during pregnancy increased the specific antibody titers in offspring.
- Immunization with OmpX and GroEL induced Th2 and Th1 immune responses, respectively.

## Introduction

*Cronobacter* spp., previously known as *Enterobacter sakazakii*, are gram-negative, rod-shaped, motile, facultative anaerobic bacteria (Farmer et al., 1980). They are a group of opportunistic pathogens that primarily infect people with low immunity, such as infants and young children (Feeney et al., 2014). Due to the strong resistance of *Cronobacter* spp. to drying, various breast milk substitutes, including common milk powder, have become a source of infection in infants and young children (Al-Nabulsi et al., 2009; Lang et al., 2017). *Cronobacter* spp. contain seven species, among which *Cronobacter sakazakii* is the most prevalent and is usually identified in foods and clinical cases (Holý and Forsythe, 2014). *C. sakazakii* can cause necrotizing enterocolitis and sepsis in neonatal intensive care unit patients. It can also penetrate the blood brain barrier and cause meningitis (van Acker et al., 2001; Gurtler et al., 2005). The mortality rate for neonatal infections has been reported to be as high as 40–80% (Lai, 2001). Therefore, improving neonatal resistance to *C. sakazakii* infection is crucial to the health of newborns.

The immune system of infants is functionally naive and immature, affecting adaptive and innate immune responses (Ofer and Wynn, 2014) and placing newborns at higher risk of infection with common pathogens. It has been reported that maternal immunity can enhance neonatal resistance to pathogens (Kumar and Bhat, 2016). Vaccinating females during pregnancy can increase levels of specific maternal antibodies that can confer protection to offspring. Studies have shown that maternal vaccination can passively transfer protection to protect offspring from deadly influenza attacks (van der Lubbe et al., 2017). The immune protection of newborns depends mainly on the passive transfer of immunoglobulin G from mothers (Kumar and Bhat, 2016). Maternal IgG is passively transferred mainly through the placenta (Blumberg et al., 1995) and breast milk (Donovan and Comstock, 2016). Furthermore, maternal immune-derived cytokines have also been proved to play an important part in the early defense against infection and immune regulation in progeny (Elahi et al., 2017). These studies provide a useful reference to improve resistance to *C. sakazakii* infections in newborns. However, to our knowledge, no studies on maternal immune protection against *C. sakazakii* have been reported.

Immunogenic bacterial proteins play vital roles in bacterium-host cell interactions and inducing host immune responses. In our previous study, GroEL was found to be a potential immunogen of *C. sakazakii* by an immunoproteomic approach (Wang et al., 2013). GroEL is a molecular chaperone and belongs to the family of heat shock proteins, which play important roles in the proper assembly and folding of proteins. In addition, molecular chaperones may be effective proinflammatory and immunomodulatory signals and can contribute to apparent immune activation (Lewthwaite et al., 1998). It has been found that immunization with GroEL can induce an immune response and reduce the degree of lesion and mortality, which provides protection against pathogenic infections with *Salmonella typhi* and *Porphyromonas gingivalis* (Bansal et al., 2010; Hagiwara et al., 2014). Many outer membrane proteins can promote the adhesion and invasion of bacteria in host cells and are important virulence factors and immunogenic proteins. A study showed that OmpX immunization conferred resistance to challenge with *Edwardsiella tarda* and induced a stronger immune response in flounder, suggesting that OmpX was a promising vaccine candidate against *E. tarda* infection (Liu et al., 2017). However, the protective effect of the two proteins against *C. sakazakii* infection has not been investigated. It is significant to explore their vaccine potentials in neonates considering that *C. sakazakii* is an important pathogen mainly threatening infants.

In this study, recombinant expression vectors containing the GroEL and OmpX genes of *C. sakazakii* were constructed, and the purified proteins were used as immunogens to immunize pregnant rats. The immunogenicity of the two recombinant proteins and the protective efficacy of maternal immunity in the offspring were evaluated. This study provides a possible strategy to help infants resist the damage caused by *C. sakazakii* infection.

## Materials and Methods

### Rats

Pregnant 4-month-old female rats, obtained from Sibeifu (Beijing, China), were given regular rat feed and water *ad libitum*. Rats were placed in mouse cages with one rat per cage and maintained in temperature-controlled clean racks with a 12-h light-dark cycle. Rat were handled and manipulated according to the guidelines of the Institute Ethics Committee.

### Bacterial Strains, Vectors, and Reagents

*Cronobacter sakazakii* ATCC 29544 stored at Tianjin University of Science and Technology was used for genomic DNA isolation. Plasmid pET-26b(+) was purchased from Novagen (WI, USA). *Escherichia coli* DH5α and BL-21 were obtained from TIANGEN (Beijing, China). *Eco*R I, *Xho* I, and T4 DNA ligase were purchased from TaKaRa (Dalian, China). KOD DNA Polymerase was purchased from TOYOBO (Shanghai, China).

### Recombinant Plasmid Construction

The strain ATCC 29544 and *E. coli* containing plasmid pET-26b(+) stored at −80°C were inoculated onto a Luria Bertani (LB) solid medium in a sterile environment using the three-streak dilution method and then the inverted plate was incubated at 37°C for 12–18 h. A single colony was transferred to 10 mL liquid medium and cultured overnight at 37°C with shaking at 200 rpm. Genomic DNA and plasmid DNA were isolated using a bacterial DNA kit from TIANGEN and a plasmid DNA mini kit (OMEGA, Guangzhou, China), respectively, according to the manufacturer's instructions. Based on the genome sequence of *C. sakazakii* ATCC 29544 (GenBank No. CP011047.1), the gene sequences encoding GroEL (CSK29544_01429) and OmpX (CSK29544_03828) were obtained. Specific primers were designed, and the two genes were amplified by polymerase chain reaction (PCR) (Table 1). PCR was performed using target genomic DNA (100 ng) as the template, dNTPs (200 μM each), specific primers (1.5 μM each) and 1 U pfu DNA polymerase in a thermocycler with the following conditions: 94°C for 2 min (initial denaturation), 98°C for 10 s (denaturation), 55°C for 30 s (annealing), and 68°C for 1 min 30 s (extension). The reaction was carried out for a total of 30 cycles. The PCR products were purified using a DNA purification and recovery kit (OMEGA, Guangzhou, China) and digested with the specific restriction enzymes *Eco*R I and *Xho* I and then ligated into the pET-26b(+) vector using T4 DNA ligase to construct recombinant plasmids. The recombinant plasmids were transformed into *E. coli* DH5α cells by heat shock method for amplification. Colony PCR, plasmid PCR [primers specific for plasmid pET-26b(+) as shown in Table 1], double enzyme digestion, and gene sequencing (Genewiz, Suzhou, China) were used for verification of the recombinant plasmids.

**Table 1:** Primers used in this study.

**Number**	**Target gene (plasmid)**	**Primer name**	**Primer sequence**
1	GroEL	GroEL–F	5′-CCG GAATTCGATGGCAGCTA AAGACGTAAAAT-3′(*Eco*R I)
2	GroEL	GroEL–R	5′-TACCCG CTCGAGCATCATAC CGCCCATACCGCCC-3′(*Xho* I)
3	OmpX	OmpX-F	5′-CCG GAATTCGATGAAAAAAAT TGCATGTCTTT-3′(*Eco*R I)
4	OmpX	OmpX-R	5′-TACCCG CTCGAGGAAGCGGT AACCCACGCCTGCG-3′(*Xho* I)
5	pET-26b (+)	T7	5′-TAATACGACTCACTAT AGGG-3′
6	pET-26b (+)	T7-term	5′-GCTAGTTATTGCTCAGCGG-3′

*The underline refers to the restriction enzyme cutting site*.

### Expression of Recombinant Proteins

For expression of the recombinant proteins, the recombinant plasmids were transformed into *E. coli* BL21 cells cultured in LB medium (with 1 mM Kana antibiotic) by heat shock method and induced with 0.05 mM isopropylthiogalactoside (IPTG). GroEL was induced with 0.05 mM IPTG for 8 h at 30°C, and OmpX was induced with 0.05 mM IPTG for 5 h at 37°C. The bacterial cells were collected after induction and disrupted at 25 MPa using a high-pressure homogenizer (Constant Systems, UK). The disrupted cells were centrifuged at 10,000 rpm and analyzed. The supernatant and pellet were analyzed by 12% acrylamide gel electrophoresis (SDS-PAGE). If the pellet was insoluble, it was denatured and renatured with urea dialysate (Shi et al., 2015).

### Purification of Recombinant Proteins

The supernatant obtained by disrupting the cells with a high pressure homogenizer was added to a protein purification column packed with Ni-NTA His-Bind Resin (Millipore, Massachusetts, USA) and pre-equilibrated with binding buffer (200 mM NaH_2_PO_4_, 1,200 mM NaCl, 40 mM imidazole, pH = 8.0). The heteroproteins were eluted using a washing buffer (200 mM NaH_2_PO_4_, 1,200 mM NaCl, 80 mM imidazole, pH = 8.0), and the desired recombinant protein was eluted using an elution buffer (200 mM NaH_2_PO_4_, 1,200 mM NaCl, 1,000 mM imidazole, pH = 8.0). The eluted components were then dialyzed using Amicon filtration columns (Millipore, Massachusetts, USA). The purification efficiency of the recombinant proteins was analyzed using SDS-PAGE. The gel was stained by coomassie blue staining solution. The concentration of the recombinant proteins was determined using a BCA protein quantification kit (Solarbio, Beijing, China). The protein solution was mixed with mannitol and sucrose to a final concentrations of 1.0 mg/mL and lyophilized. The lyophilized proteins were stored at −20°C for further use.

### Immunization of Pregnant Rats and Challenge of Offspring

**Figure 1:**
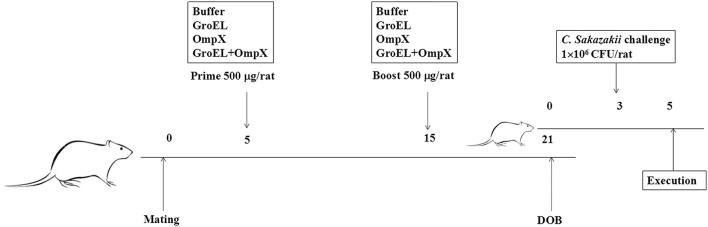
The schedule of pregnant rat immunization and offspring rat infection. Pregnant rats were randomly divided into four groups (*n* = 5/group) for immunization. Subcutaneous injection of recombinant protein was performed at the fifth and fifteenth days of gestation. The rats were infected with *C. sakazakii* ATCC 29544 (100 μL of 1 ×10^7^ CFU/mL bacterial cells mixed with 100 μL sterilized goat milk powder) on the third day after birth, and the rats were sacrificed on the fifth day after birth.

Pregnant rats were randomly divided into four groups (five rats in each group) for immunization. Male and female rats were caged, and the female rats with a vagina plug were defined as the first 0 day of pregnancy. Subcutaneous injection of recombinant protein on the back of the rat was performed at 5 and 15 days of gestation. The first three groups were initially immunized with 500 μL of 1 mg/mL recombinant protein (GroEL, OmpX, GroEL+OmpX) and an equal volume of complete Freund's adjuvant. Then, 500 μL of 1.0 mg/mL recombinant protein (GroEL, OmpX, GroEL+OmpX) and an equal amount of incomplete Freund's adjuvant were used for booster immunization. The fourth group was initially immunized and boosted with 500 μL of PBS solution mixed with an equal amount of complete Freund's adjuvant and incomplete Freund's adjuvant, respectively. The offspring from each group were randomly divided into a gavage group and a blank control group. Bacterial challenge experiments were performed on offspring at 3 days of age. The rats were infected by intragastrical gavage with *C. sakazakii* ATCC 29544 (100 μL 1×10^7^ CFU/mL ATCC 29544 mixed with 100 μL sterilized goat milk powder), and 0.9% NaCl (100 μL 0.9% NaCl solution mixed with 100 μL sterilized goat milk powder) was used as a blank control. The infected rats were separated from the mother. During the course of the experiment, the offspring were fed with 50 μL of sterile goat milk powder every 4 h. The symptoms were observed, and the number of death were recorded at regular intervals. All infected rats were sacrificed at 42 h after infection (Figure 1).

### Safety Assessment of Pregnancy

The weights of the pregnant rats were recorded every 3 days during the feeding process. The length of pregnancy for each pregnant rat was recorded. After the birth of the offspring, the number of litters per pregnant rat and the weight of each offspring were recorded.

### Recombinant Protein-Mediated Bacterial Clearance

The bacterial challenge experiment was carried out when the offspring were 3 days old. Rats were sacrificed 2 days after the challenge with 1 × 10^6^ CFU/rat of *C. Sakazakii* ATCC 29544 cells. Then, brain and blood samples were collected in a sterile environment to analyze the bacterial infection. The brain tissue was placed in 1.0 mL sterile PBS, and the samples were fully homogenized and inoculated in modified lauryl sulfate tryptose vancomycin medium (mlst-vm medium). The blood was diluted 10 times with sterile PBS and inoculated in mlst-vm medium. After incubation for 24 h in a 37°C incubator, colony counts were determined.

### Histology Analysis

For histological examination, brain and small intestine were collected 2 days after infection, fixed in 4% paraformaldehyde, embedded in paraffin, and serially sectioned. Serial 5-μm tissue sections were subjected to hematoxylin-eosin (HE) staining and examined under a light microscope (HAMAMATSU, Japan).

### Determination of Specific Antibody Titers

The mothers were sacrificed when the offspring were separated, and blood was collected from the thigh femoral artery. The offspring were sacrificed by breaking the neck, and blood was collected at the age of 5 days. The blood was centrifuged at 6,000 rpm for 5 min, and the supernatant was collected and stored at −80°C until analysis. Specific antibody titers were determined by enzyme-linked immunosorbent assay (ELISA). Briefly, 0.1 mg/mL recombinant protein (GroEL, OmpX, or GroEL+OmpX) diluted in coating buffer (15 mM Na_2_CO_3_, 35 mM NaHCO_3_ pH 9.6) was added to a 96-well microtiter plate and incubated overnight at 4°C. On the next day, each well was blocked with PBS containing 0.5% skim milk powder for 1 h at 37°C, following by incubating with serially diluted serum samples for antibody detection. Each well was washed three times with washing buffer between each step and patted dry. Then, 100 μL of goat anti-mouse IgG-HRP (1:10,000 dilution) was added and incubated at 37°C for 30 min. Next, 100 μL of freshly prepared TMB substrate solution was added and the microplates were incubated at 37°C for 10 min for colorimetric detection. The reaction was terminated with 50 μL/well of 12 M NaOH, and the absorbance was measured at 450 nm using a microplate reader.

### Detection of Cytokines

The offspring were sacrificed by breaking the neck, and blood was collected at the age of 5 days. The blood was centrifuged at 6,000 rpm for 5 min, and the supernatant was collected and stored at −80°C until analysis. The levels of IL-4 and IFN-γ in the serum of offspring were measured using ELISA kits (BIOSWAMP, Shanghai, China) according to the manufacturer's instructions. The absorbance was read at 450 nm using a microplate reader (Thermo Fisher Scientific, Massachusetts, USA).

### Statistical Analysis

Statistical analysis was performed using SPSS 19.0 and Origin 8.0 software. Differences among groups were analyzed by one applying one-way analysis of variance (ANOVA) and Mann–Whitney unpaired non-parametric *t*-test. Data were expressed as the means ± standard deviation (SD). The differences were considered significant at *p* < 0.05 (Supplementary Data Sheet 2).

## Results

### Expression and Purification of Recombinant Protein

**Figure 2:**
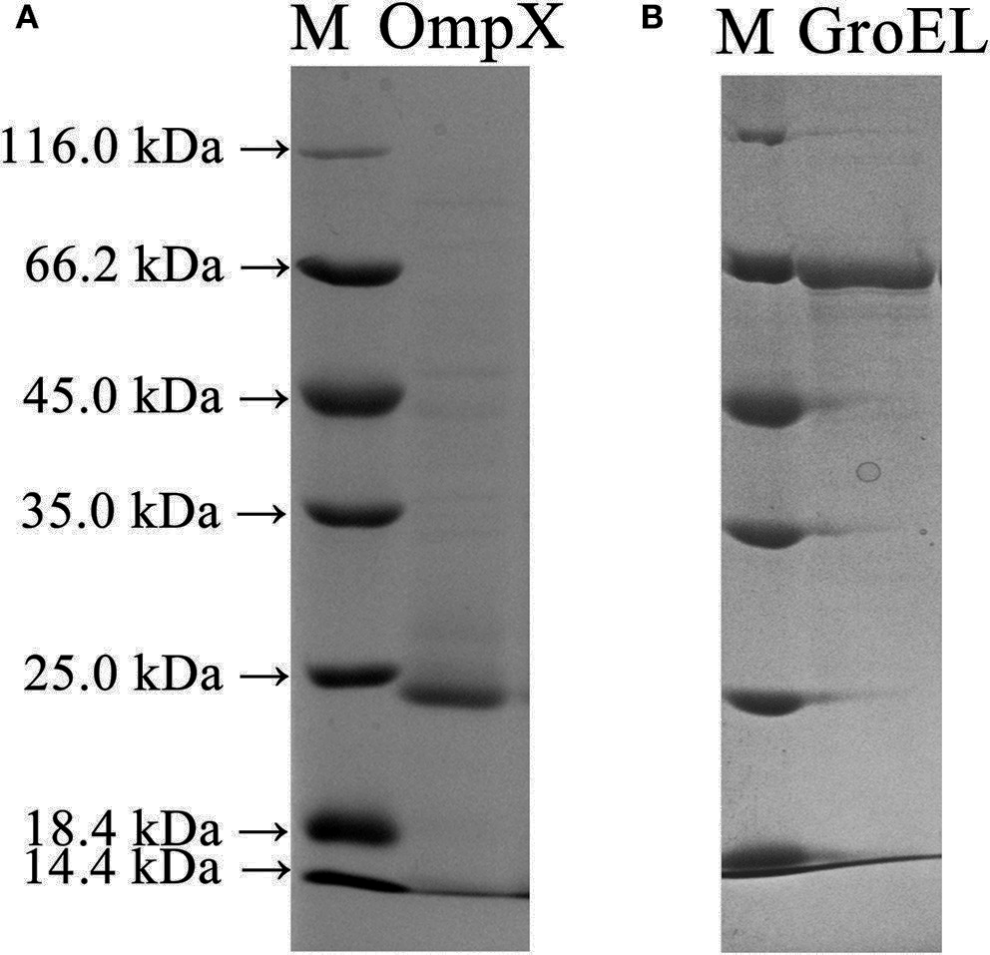
SDS-PAGE profile depicting expression and purification of the OmpX and GroEL proteins in *E. coli* BL21 (DE3) cells. **(A)** The recombinant OmpX protein purified by Ni-affinity chromatography. **(B)** The recombinant GroEL protein purified by Ni-affinity chromatography.

The full-length genes of GroEL and OmpX were amplified from the genomic DNA of *C. sakazakii* ATCC 29544 and ligated into the pET-26b(+) vector to construct recombinant plasmids. The recombinant plasmids were introduced into *E. coli* BL21 cells to express the recombinant proteins. SDS-PAGE analysis showed that GroEL and OmpX were well-expressed when the cells were induced by 0.05 mM IPTG. The recombinant protein GroEL was expressed in a soluble form, and the recombinant protein OmpX was mainly expressed in the form of inclusion body (Figure S1). After denaturation and renaturation by urea, the OmpX inclusion body was converted to a soluble form (Figure S2). The recombinant proteins were purified by a Ni-affinity chromatography column. SDS-PAGE analysis of the purified proteins revealed a single band of approximately 60 and 20 kDa for GroEL and OmpX, respectively (Figure 2).

### Safety Assessment of Pregnancy

**Figure 3:**
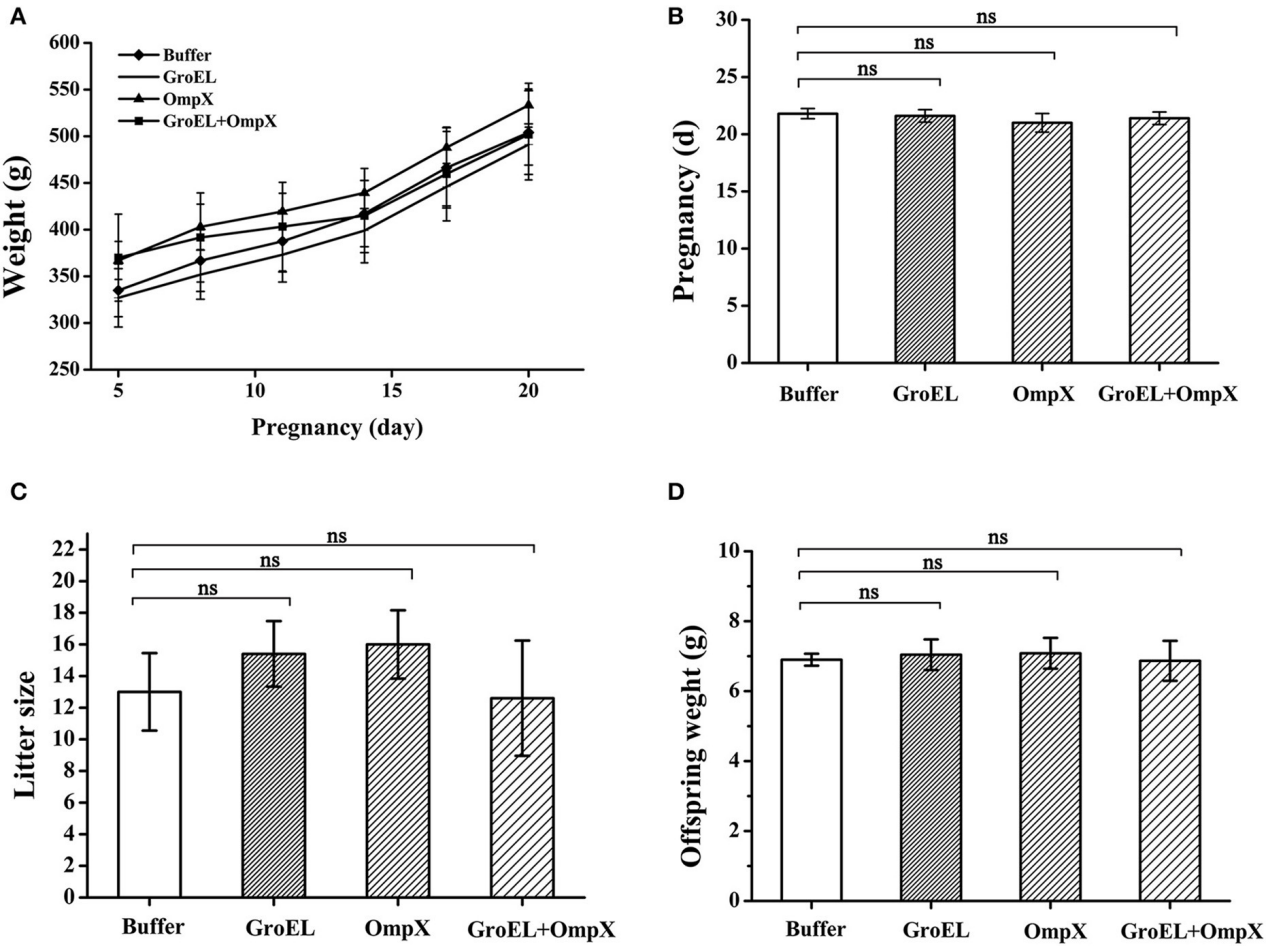
Safety assessment of immunization with recombinant proteins. **(A)** The weight of each pregnant rat in each group was recorded every 3 days. **(B)** Pregnancy duration for each rat was recorded. **(C)** The offspring number of each pregnant rat was recorded. **(D)** Weight of each offspring was recorded. The results are expressed as the mean ± SD of each group. There are all five pregnant rats in each group except only four rats in the OmpX group. Because the OmpX group removed one rat which failed to conceive. The differences were considered significant at *p* < 0.05. *, **, *** represent *p* < 0.05, *p* < 0.01 and *p* < 0.001, respectively (compared with the control group). Data were analyzed with Mann–Whitney unpaired non-parametric *t*-test.

The weights of pregnant rats were recorded every 3 days during the breeding process. The results showed that the pregnant rats immunized with the recombinant proteins had a highly similar weight change to the blank controls, and both groups showed a steady upward trend (Figure 3A). The length of pregnancy of each pregnant animal was recorded, and the results showed that the pregnancies of the rats immunized with the recombinant proteins were almost identical in duration compared to the control (Figure 3B). After birth, the number of offspring of each pregnant rat and the weight of each offspring were recorded. The results showed that there was no significant difference in the number and weight of offspring between the recombinant protein-immunized groups and the control group (Figures 3C,D). These results indicated that the recombinant proteins GroEL and OmpX and the mixture of these two proteins had no adverse effect on the weight gain, duration of pregnancy, litter size, and weight of the offspring.

### Challenge Studies

**Figure 4:**
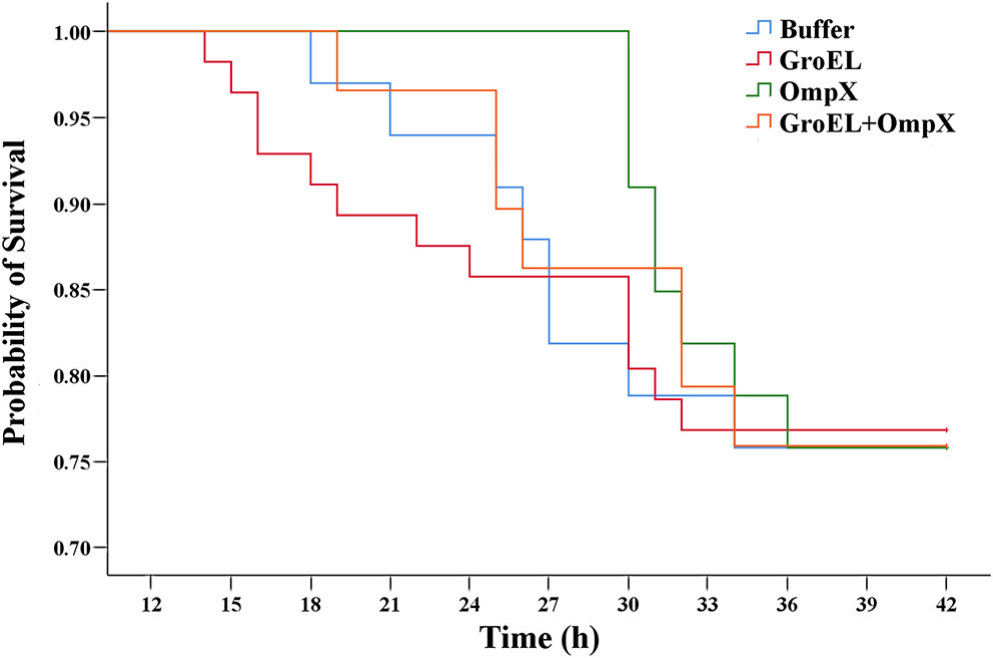
Probability of survival of offspring after bacterial infection. Offspring were challenged with 100 μL of 1 ×10^7^ CFU/mL *C. sakazakii* ATCC 29544 by gavage. The death rate in each group was recorded every 3 h. Control group: *n* = 33 rats; GroEL-immunized group: *n* = 56 rats; OmpX-immunized group: *n* = 33 rats; GroEL+OmpX-immunized group: *n* = 29 rats. Time of death of pups that died after infection were analyzed with log-rank test and the result showed that the OmpX group exhibited significant delayed death occurrence (*p* = 0.025).

To assess the immunoprotective effect of the recombinant proteins, bacterial challenge experiments were performed at 3 days after birth, and mortality was monitored. The results showed that although the mortality rate of each group was almost at the same level after bacterial infection, the time from challenge to death of each group was different. Compared to the blank control group, the time to death and mortality of the group immunized with recombinant protein GroEL was similar. In comparison with the control group, although the final mortality of the OmpX-immunized group and the GroEL+OmpX-immunized group was almost the same, offspring death was delayed by immunization with the recombinant proteins. Particularly, in the OmpX-immunized group, death was delayed by nearly 12 h in comparison with the control group (Figure 4). Therefore, according to the time to death and mortality rates after infection, among the three proteins, OmpX exhibited the best protection effect against *C. sakazakii* infection, followed by the GroEL+OmpX mixture.

### Recombinant Protein-Mediated Bacterial Clearance

**Figure 5:**
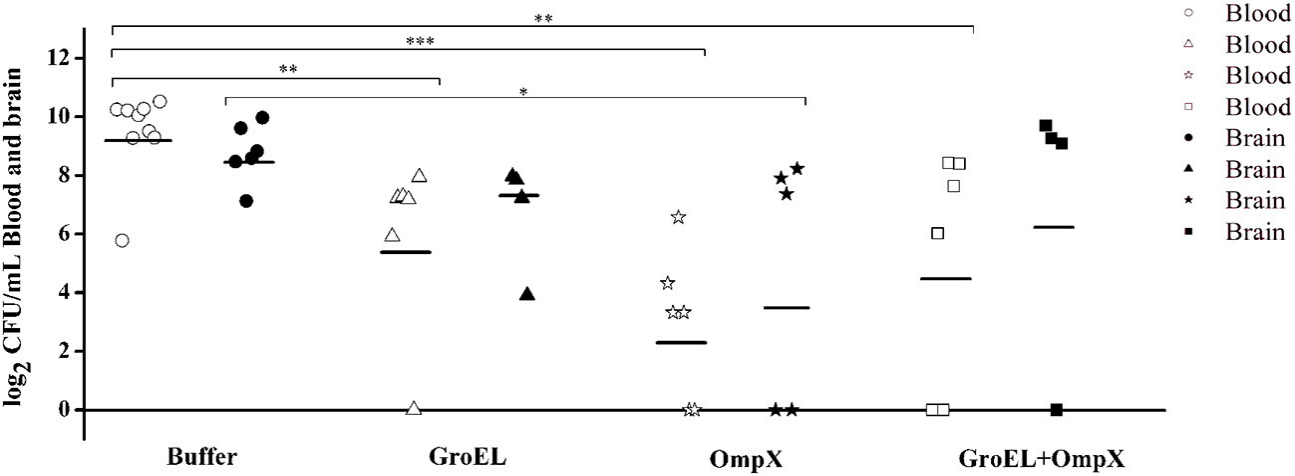
Bacterial amount detected in the blood and brain of *C. sakazakii* ATCC 29544-challenged offspring. Each dot represents one animal. The horizontal lines indicate the average CFU per blood or brain. The differences were considered significant at *p* < 0.05. *, **, *** represent *p* < 0.05, *p* < 0.01 and *p* < 0.001, respectively. Data were analyzed with ANOVA.

To analyze the recombinant protein-mediated protection, the bacterial load in blood and brain in the offspring was determined after challenge with ATCC 29544. The results showed that the bacterial load in the blood and brain of the baby rats decreased after inoculating the recombinant protein compared with that of the mother rats inoculated with PBS. Furthermore, the bacterial load in the blood of the immunized groups was generally lower than that in the brain (Figure 5).

### Analysis of Lesion Area

**Figure 6:**
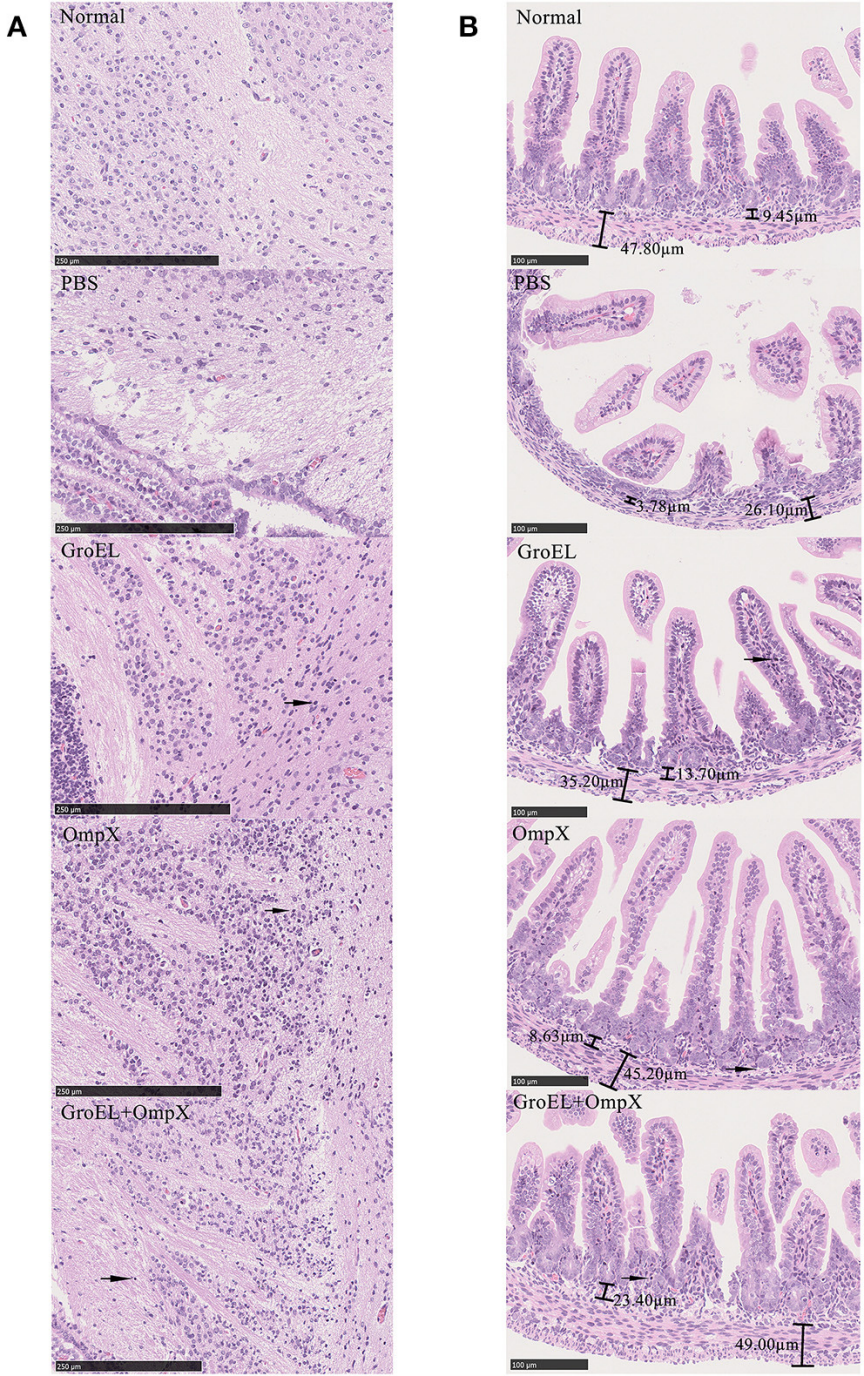
Analysis of brain and intestine tissue. **(A)** Pathological analysis of brain tissue after bacterial challenge in groups immunized with PBS, GroEL, OmpX, or GroEL+OmpX. **(B)** Pathological analysis of the intestinal tissues after bacterial challenge in groups immunized with PBS, GroEL, OmpX, or GroEL+OmpX. Inflammatory cells are marked with arrows.

To analyze the histological changes in the progeny of each group after bacterial challenge, the brain and small intestine tissues of the offspring were collected on the second day after infection and processed for HE staining. Meningitis can lead to a loose structure of the brain matrix. Enteritis can cause villi of intestinal tissue edema, fall off, arrange disorder, crypts atrophy, and bowel wall thinning. Compared with that in normal brain tissue, the matrix in the offspring brain tissue of the control group (immunized with PBS) was dissolved and loose, presenting a sponge-like structure after bacterial challenge (Figure 6A). In the GroEL-immunized group and the GroEL+OmpX-immunized group, the cerebral matrix was slightly loose, and inflammatory cell infiltration was observed (Figure 6A). The OmpX-immunized group had normal brain matrix structure and a large area of inflammatory cell infiltration (Figure 6A). Through analysis of the structure of intestinal tissue, it was found that the normal intestinal tissue had dense and evenly arranged villi, and the submucosal and muscular layers could be clearly defined and were thicker (Figure 6B). In the PBS-immunized group, villi were rare and shed, crypt had atrophied and intestinal wall became thin. Individual villi of the GroEL group appeared to fall off, the base was slightly thinned, and a small amount of inflammatory cells appeared (Figure 6B). In the OmpX group, the villi were arranged neatly and tightly without edema, with deep crypts and increased inflammatory cell infiltration. The histological morphology was similar to that of the normal group. In the GroEL+OmpX group, the villi were arranged in an orderly manner but shed. The depth of the crypt became shallow, and inflammatory cells appeared.

### Specific Antibody Response to Recombinant Protein Vaccination

**Figure 7:**
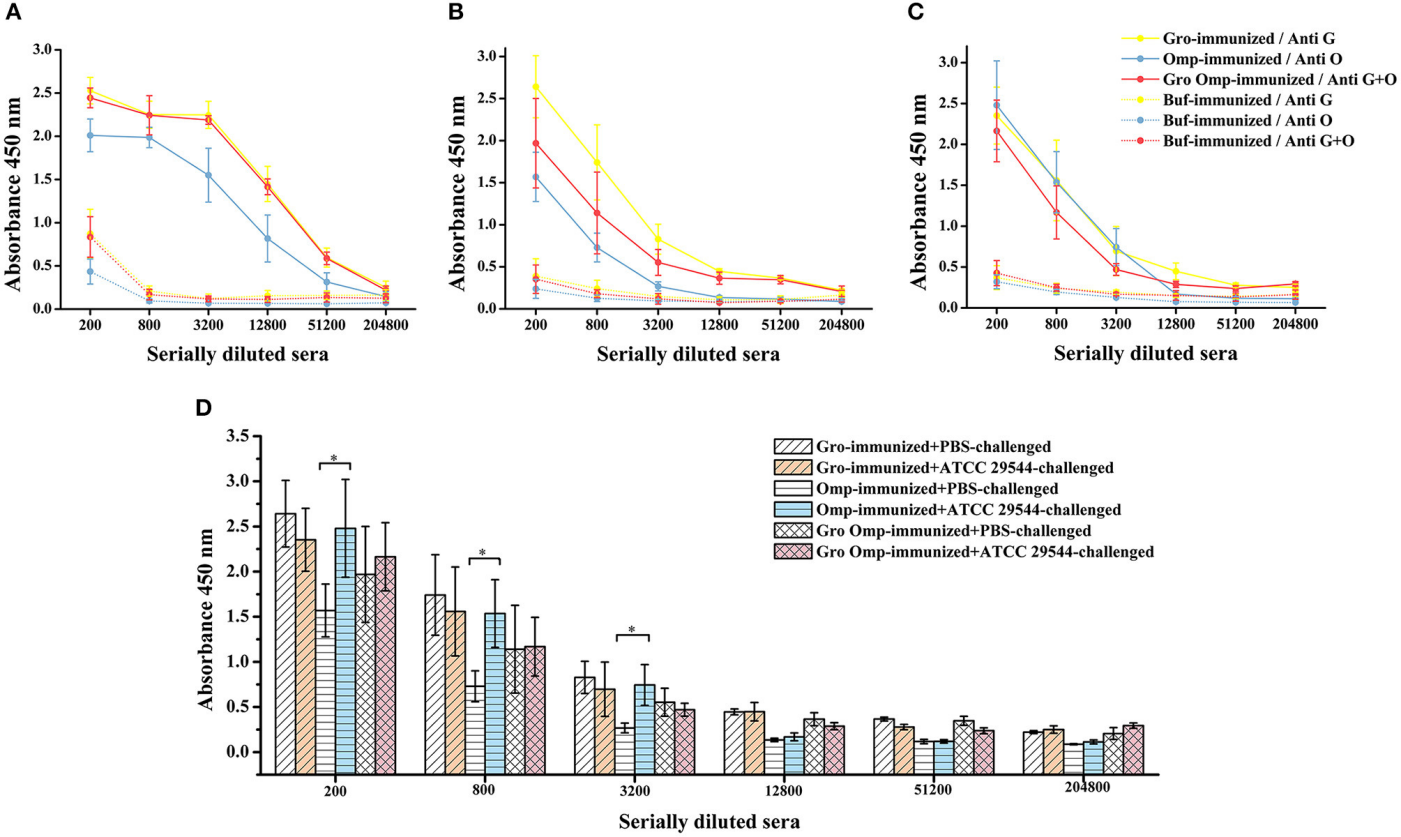
Effect of recombinant protein immunization on specific antibody titers in mother and offspring. Varying dilutions of immune sera were assayed for IgG with recombinant protein. **(A)** Levels of specific antibodies in the protein-immunized pregnant rats and control pregnant rats. **(B)** Levels of specific antibodies in the progeny of the protein-immunized and blank control pregnant rats. **(C)** Levels of specific antibodies in the serum of the offspring after *C. sakazakii* ATCC 29544 challenge for 2 days. **(D)** Comparison of serum-specific antibody levels in offspring challenged with *C. sakazakii* ATCC 29544 and PBS. *n* = 5 rats per group. The differences were considered significant at *p* < 0.05. *, **, *** represent *p* < 0.05, *p* < 0.01 and *p* < 0.001, respectively (compared with control group). Data were analyzed with Mann–Whitney unpaired non-parametric *t*-test.

To determine the antigen-specific humoral responses primed by immunization with the recombinant proteins, specific antibodies in the serum of pregnant rats and offspring were detected by indirect ELISA. In general, antibody levels in pregnant rats immunized with the recombinant proteins increased significantly compared to those in the control group. The pregnant rats inoculated with recombinant proteins showed significantly higher anti-GroEL, anti-OmpX, and anti-GroEL+ OmpX IgG titers than the control group (Figure 7A). The offspring of the immune groups also had significantly higher levels of specific IgG compared to the blank control group (Figure 7B). The results showed that these three sets of recombinant proteins induced a specific humoral response in the mother, and the specific antibodies produced were also transferred to the progeny passively. After bacterial challenge, the immune groups had higher levels of specific antibody titers compared to the group immunized with PBS, which was also challenged with *C. sakazakii* (Figure 7C). Compared with that of the other two immune groups, the specific antibody titer after bacterial challenge in the OmpX group was significantly higher than in the group which injected with PBS instead of *C. sakazakii* (Figure 7D).

### Effect of Recombinant Protein Immunization on Cytokine Production

**Figure 8:**
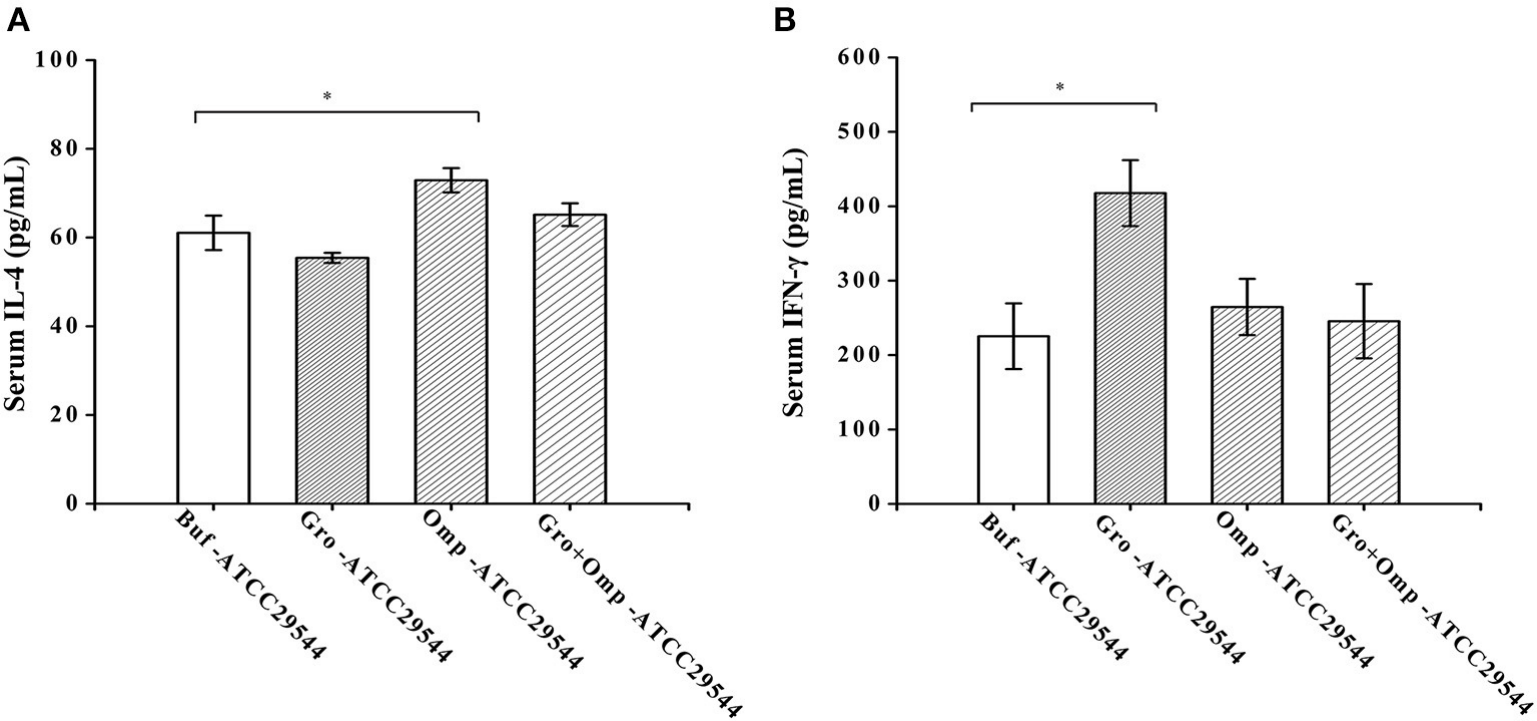
Effect of recombinant protein immunization on the production of IL-4 and IFN-γ in the offspring. **(A)** Levels of IL-4 in the serum of the offspring after challenge. **(B)** Levels of IFN-γ in the serum of the offspring after challenge. Three-day-old offspring were challenged with ATCC 29544, and serum was collected 36 h later. The levels of IL-4 and IFN-γ were assessed by cytokine-specific ELISAs. *n* = 4 rats per group. The differences were considered significant at *p* < 0.05. *, **, *** represent *p* < 0.05, *p* < 0.01 and *p* < 0.001, respectively (compared with the control group). Data were analyzed with Mann–Whitney unpaired non-parametric *t*-test.

To investigate the effects of maternal immunity on cytokine levels in the offspring and the impact of the recombinant protein on the regulation of cytokines, the production of Th1-type (IFN-γ) and Th2-type (IL-4) cytokines were detected in the serum of the offspring. Compared to the control group, the offspring of the GroEL-immunized group and the OmpX-immunized group produced significantly higher levels of IFN-γ and IL-4 after challenge with *C. sakazakii* (Figures 8A,B).

## Discussion

*C. sakazakii* is a common foodborne pathogen that mainly causes life-threatening infections in newborns. Although neonates can overcome the high morbidity and mortality of early infection by immunization (Saso and Kampmann, 2017), this method of protection in neonates is less immunogenic and safe. In humans, maternal IgG can be actively transferred to the fetus through the Fcγ receptor (Hanson et al., 2010; Voysey et al., 2017), which usually results in approximately 90% of the maternal serum level of IgG antibodies present in a full-term newborn at delivery. This is important to provide protection against infections during the first few months of life. Therefore, passive immunization methods can be considered to provide rapid, timely and effective protection for newborns. However, to our knowledge, no studies on passive immune protection against *C. sakazakii* have been reported. In this study, two potential immunogens (GroEL and OmpX) were used to immunize pregnant rats, and the protective effect on the offspring was evaluated and investigated. The findings of this study provide a useful reference for improving the resistance of infants to *C. sakazakii* infection.

Many protein candidates located on the bacterial surface may have protective immune properties. GroEL, a member of the heat shock protein (Hsp) family, is a highly conserved, essential molecular chaperone expressed in eukaryotes and prokaryotes that is extensively expressed in a variety of bacteria. In addition to assisting the folding or unfolding and the assembly or disassembly of other macromolecules, chaperones of both mammalian and microbial origin can activate a variety of cell populations directly (monocytes, macrophages, and dendritic cells) and stimulate them to produce nitric oxide and various cytokines (Marcatili et al., 1997; Wallin et al., 2002). GroEL has also been found to regulate the production of PTX3, a soluble form of pattern recognition receptor (Shin et al., 2016). The activation of various cell populations and the regulation of PTX3 by GroEL may lead to potent proinflammatory and immunomodulatory effects of the chaperone, so the Hsp has the dual role of a molecular chaperone and a cytokine. In our previous studies, GroEL showed high expression levels and strong immunogenicity (Wang et al., 2013). OmpX is an outer membrane protein that is essential for the basal invasion of *C. sakazakii* and can help the bacterium translocate into the deeper organs (spleen and liver) of rats (Singh et al., 2015), suggesting the key roles of OmpX associated with *C. sakazakii* virulence. PCR analysis showed that the OmpX gene was present in all 164 tested *C. sakazakii* strains and 16 tested *C. malonaticus* strains, which are the two main species associated with clinical cases (Eibach et al., 2014). Two-dimensional gel electrophoresis (2-DE) analysis showed that OmpX was highly expressed in a virulent *C. sakazakii* isolate (Ye et al., 2015). These two proteins showed a high expression level and strong immunogenicity or key role in virulence, suggesting their significant potential as vaccine candidates and were, therefore, selected in this study.

To date, there are no animal experiments on the immune properties of these two proteins in *C. sakazakii*. To assess the immunoprotective effect accurately. In this study, GroEL and OmpX were recombinantly prepared as antigens to immunize rats, and their potential use as maternal vaccines for passive immunization of offspring were evaluated. Bacterial challenge in suckling rats showed that immunization with OmpX recombinant protein and GroEL+OmpX recombinant protein delayed the occurrence of offspring death, and the OmpX group had the most significant protective effect. However, although the GroEL group did not show a delay in offspring death, the bacterial colony counts in the blood and brain showed that all three groups immunized with recombinant proteins reduced the bacterial load in the offspring. Some individuals in the immune groups has undetectable bacteria in the brain. Reducing the burden of pathogens means increased bacterial clearance and reduced bacterial reproduction, thereby weakening of meningitis and bacteremia and thus delaying death. Compared with the experimental groups, the progeny of the group immunized with PBS mostly had neck stiffness, head backwards, back stiffness, and the whole body bent back like a “bow” before death, which are typical symptoms of meningitis. Besides, it was found that the blood of the protein-immunized groups had a lower bacterial load than the brain, which may be caused by the clearance of bacteria by antibodies in the blood. By HE staining of the offspring's brain and intestinal tissues, it was found that immunization with the recombinant proteins in the mother increased inflammatory cell infiltration and reduced tissue damage in the offspring after bacterial challenge. Furthermore, immunization with the recombinant proteins did not affect the development of pregnant rats or offspring during pregnancy, indicating the safety of immunization with the recombinant proteins. This study first proved that GroEL and OmpX were effective in inducing the immune response in pregnant rats and newborns against *C. sakazakii* infection and were safe for pregnant mothers and their offspring.

Offspring obtain IgG from the mother through the placenta or breast milk. The binding of IgG to the cell surface FcγR contributes to the immune response directly, granting IgG an important role in immunity, especially in the memory immune response. IgG can bind and neutralize antigens independently and coat microorganisms to promote phagocytosis to achieve antibacterial effects (Schroeder and Cavacini, 2010). Compared with the control group, the progeny of the protein-immunized groups had higher levels of cytokines (Figures 8A,B). The cytokines in the progeny serum may be mainly derived from the absorption of maternal cytokines in the colostrum (Hsueh et al., 2016). Passive transfer of cytokines and antibodies further affects the cytokine secretion capacity of neonatal spleen cells (Elahi et al., 2017). By analyzing the cytokines in the serum of the progeny after bacterial challenge, we found that the GroEL group produced more IFN-γ, mainly shifting to Th1-type immunity, which was similar to previous reports (Sophie et al., 2002). The OmpX group produced more IL-4, indicating that Th2-type immunity was activated. IFN-γ can increase the phagocytosis of antigen-presenting cells, and IL-4 can increase the ability of B cells to present antigen and has high immunogenicity. As a typical outer membrane protein, OmpX is widely expressed in bacteria. However, its vaccinal properties and underlying mechanism have been studied in few microorganisms. In *E. coli*, recombinant OmpX could induce a Th1/Th2 mixed immune response (Maisnier-Patin et al., 2003). In *E. tarda*, OmpX elicited a significantly higher immune response than most other outer membrane proteins (Sophie et al., 2002; Liu et al., 2017). Interestingly, as a chaperone protein, GroEL showed significant vaccine potential in many bacterial pathogens, such as *Streptococcus pneumonia* (Mohammed Nadeem et al., 2006), *Bacillus anthracis* (Sinha and Bhatnagar, 2010) and *E. tarda* (Liu et al., 2016). However, in this study, although GroEL of *C. sakazakii* induced a significant immune response at the molecular level, the bacterium-challenged offspring of the GroEL-immunized pregnant rats did not exhibit advantages in terms of the time of death and death rate in comparison with the PBS-immunized group. Compared to OmpX, GroEL showed weaker protective efficiency, which was not in agreement with its high expression level and immunogenicity. A previous study also showed that GroEL did not produce a significant level of protection, although it induced an immune response (Sophie et al., 2002). This contradiction may be due to the location of this protein. In gram-positive bacteria, GroEL was proven to be a surface protein (Li et al., 2019). However, in gram-negative bacteria, it was identified in both the outer membrane and the secretome (Liu et al., 2016). It is reasonable to speculate that the different locations of this protein decrease its immune protective effect, considering that the location of GroEL has not been determined in *C. sakazakii*. The mixed immunity of GroEL and OmpX did not produce a better protection effect than the immunity of OmpX alone, this was most likely because the immune amount of the OmpX in the mixed immunization group was only half of that of OmpX group. The deficiency of protein immune may be the cause of the weakened protective effect.

According to the current knowledge, the neonatal immune system is immature. Therefore, upon bacterial exposure, the specific antibodies transferred from the mother are used to provide protection resulting in the decrease in antibody in the progeny. Interestingly, compared with that in the other two immune groups, the titer of specific antibodies after bacterial challenge in the OmpX group was significantly higher than that in the PBS group (Figure 7D). There are several possible explanations for this observed difference. First, during pregnancy, the immune responses of B cells and T cells *in utero* occur after maternal vaccination, which can induce an active immune response in the fetus *in utero* and produce immune memory (Deepa et al., 2007; Vanderbeeken et al., 2013). Upon stimulation by the same antigen, a large number of specific antibodies are rapidly produced. Second, the neonatal immune system is not immunodeficient (Kollmann et al., 2017), but the initial setting of the antigen threshold required to initiate an immune response is higher than in adults. Once this threshold is reached, a considerable immune response is generated (Ghazal et al., 2013). However, with regard to passive immunization, higher maternal antibody levels have an adverse effect on the production of specific antibodies in the offspring. This is because a large number of maternal antibodies can rapidly neutralize antigens, prevent their replication and lead to a low antigenic load, and thus fail to allow infant B cells and T cells to produce antigen-specific antibodies (Albrecht et al., 1977). In the current study, the progeny of the OmpX group obtained fewer specific antibodies from the mother (Figure 7B); therefore, fewer antigens were neutralized in the face of challenge, which allowed for more efficient antigen uptake and presentation, leading to higher levels of specific antibodies.

In summary, this study suggested that recombinant protein immunization during pregnancy was beneficial to newborns respond to infections of *C. sakazakii*, and maternal immunization provides effective protection for young infants who could not be vaccinated for variety reasons. In the future, pregnant women could have appropriate exposure to pathogens or their antigens so that infants can obtain better immunity against bacterial exposure. This study provides a reference and a possible strategy to improve the ability of newborns to resist harm caused by *C. sakazakii* infection.

## Data Availability Statement

All datasets generated for this study are included in the article/Supplementary Material.

## Ethics Statement

The animal study was reviewed and approved by Institute Ethics Committee.

## Author Contributions

All the authors contributed to the writing of this article. XD and SW contributed to the conception and the design of the study. JS performed the experiments, and the writing and editing of the manuscript. YF contributed to the experimental operation. PL, TD, and XD contributed to the design and conception. All authors read and approved the final manuscript.

### Conflict of Interest

The authors declare that the research was conducted in the absence of any commercial or financial relationships that could be construed as a potential conflict of interest.
