# Supplementary material for: Protective Effect of Recombinant Proteins of Cronobacter Sakazakii During Pregnancy on the Offspring
Source: Front Cell Infect Microbiol. 2020 Jan 31;10:15. doi: 10.3389/fcimb.2020.00015 (PMC7006456; doi:10.3389/fcimb.2020.00015)
Supplement: Supplementary file 1 [file Data_Sheet_1.docx]

**
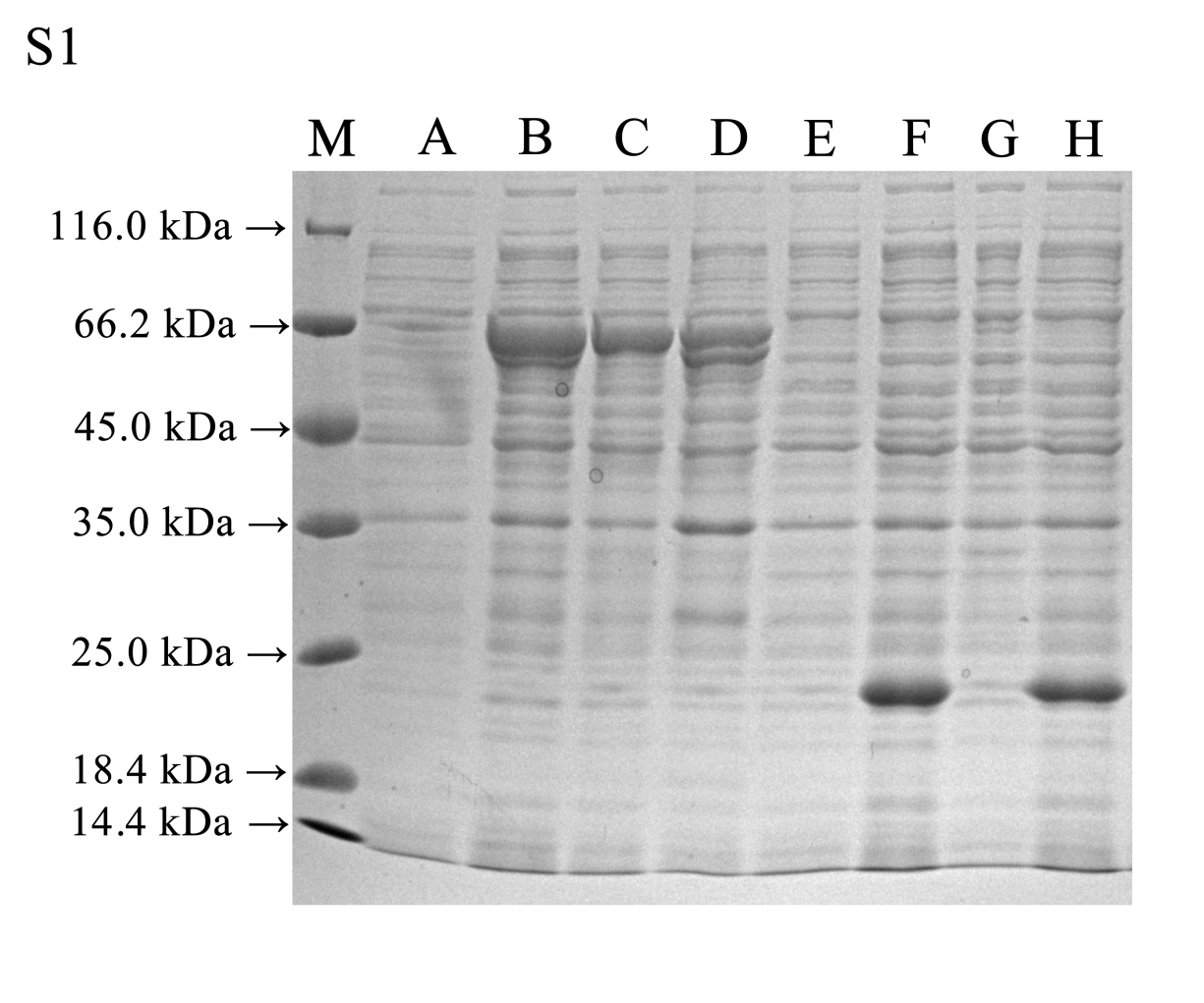
**

**Figure S1** Determination of soluble expression of the GroEL and OmpX proteins. **(M)** Molecular mass marker; **(A)** Uninduced cells expressing GroEL; **(B)** IPTG-induced cells expressing GroEL; **(C)** The supernatant obtained by disrupting the cells expressing GroEL; **(D)** The pellet obtained by disrupting the cells expressing GroEL; **(E)** Uninduced cells expressing OmpX; **(F)** IPTG-induced cells expressing OmpX; **(G)** The supernatant obtained by disrupting the cells expressing OmpX; **(H)** The pellet obtained by disrupting the cells expressing OmpX.

**
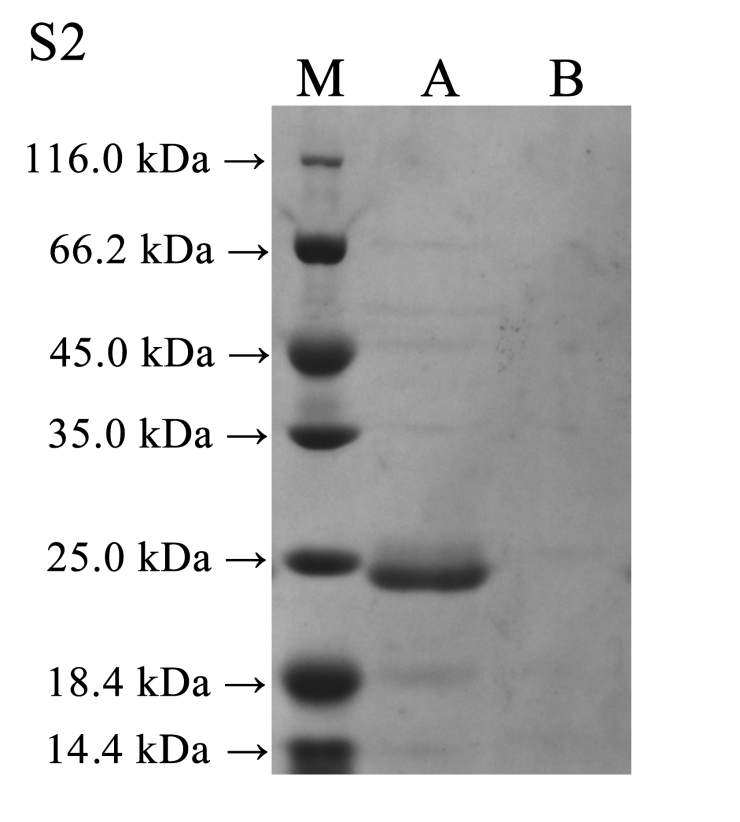
**

**Figure S2** The OmpX protein after renaturation. **(M)** Molecular mass marker; **(A)** The supernatant obtained after renaturation; **(B)** Pellet after renaturation.
